# Differential seedling responses of chickpea varieties to hexavalent chromium (VI) stress under controlled conditions

**DOI:** 10.1371/journal.pone.0341546

**Published:** 2026-01-29

**Authors:** Muhammad Hammad, Afifa Kainat Rani, Laraib Chouhdary, Muhammad Kabir, Patricio R. De los Rios-Escalante, Muhammad Aslam

**Affiliations:** 1 Department of Biological Sciences, Thal University Bhakkar, Bhakkar, Punjab, Pakistan; 2 Environment Protection and Climate Change Department, Punjab, Pakistan; 3 Department of Botany, University of Agriculture, Faisalabad, Punjab, Pakistan; 4 Department of Biological and Chemical Sciences, Faculty of Natural Resources, Catholic University of Temuco, Temuco, Chile; 5 Arid Zone Research Institute, Bhakkar, Punjab, Pakistan; University of Education, PAKISTAN

## Abstract

Hexavalent chromium (Cr VI) contamination infiltration in soil due to industrial activities has become a sever threat to Pakistan’s agricultural productivity. Recent surveys report soil chromium concentrations in agricultural–industrial interfaces of Punjab and Khyber Pakhtunkhwa reaching 100 mg/kg, exceeding the WHO permissible limit (1.5 mg/kg) by more than 50-fold. Chickpea, a widely cultivated crop in Pakistan also faces productivity threats from Cr (VI). This study evaluates the differential seedling responses of two Pakistani chickpea varieties (CM-72 and CM-98) to hexavalent chromium stress (0µM to 100µM) under controlled conditions to address a critical research gap. A fully randomized experimental design (CRD) with five replicates per treatment was utilized, assessing germination metrics, root and shoot lengths, root collar diameter, and biomass over a period of 14 days. Statistical evaluations (ANOVA, Pearson correlation, PCA) indicated substantial varietal differences. Both varieties showed significant (p < 0.05) dose-dependent reductions in germination rates, growth, and biomass; however, CM-72 exhibited greater tolerance. At a concentration of 100 µM Cr VI, CM-72 achieved 90% germination (compared to 80% in CM-98), with moderate declines in root length (39% compared to 77% in CM-98), shoot length (76% compared to 90%), and biomass (34% dry weight loss in comparison to 59% in CM-98). Multivariate analysis revealed strong associations between growth inhibition and chromium stress, with PCA differentiating between structural (root collar diameter) and temporal (germination delay) effects. These results highlight the resilience of CM-72, likely attributable to metabolic and antioxidative adaptations, making it a potential candidate for cultivation in areas contaminated with chromium. The relative tolerance of CM-72 may relate to underlying physiological or biochemical traits that warrant investigation in future studies. This research offers essential insights into the responses of chickpea varieties to Cr VI stress, highlighting the necessity for biochemical and molecular studies to clarify tolerance mechanisms and support sustainable agricultural practices in regions impacted by heavy metals.

## Introduction

Heavy metal (HM) pollution, driven by industrialization and inadequate controlling of industrial waste, is one of the foremost environmental issues worldwide, leading to increased HM contamination of farmland [1]. HMs typically infiltrate the food chain by the uptake and accumulation by plants, and subsequently passed to consumers, which can result in various health problems [2–5]. Among the various HMs, chromium (Cr) is responsible for significant soil, sediment, and groundwater pollution on a global scale [6]. It originates in the soil mainly from natural sources, including chromite deposits and volcanic activity, but can also be introduced through human activities that produce chromium-containing waste, mining operations, the disposal of electrical devices, and atmospheric deposition [7]. In Pakistan, due to industrial effluents from tanneries, textiles and electroplating units, Cr contamination has become a serious environmental and health issue [8]. Unchecked discharge of hexavalent chromium Cr (VI) in water bodies like Ravi and Chenab leads to the severe bioaccumulation of highly toxic and mobile form Cr particles in agricultural soil, particularly in Punjab and Sindh [9]. Investigations revealed the alarming and exceeding level of Cr in these regions to normal (0.05 mg/L for water and 1.5 mg/kg for soil), which leads towards the disturbed crop productivity as contaminated water irrigation causes stunt growth in crops and oxidative stress in plants due to disrupted nutrient uptake [10].

Additionally, the deposition of chromium (Cr) in agricultural soils is a worldwide issue since it is not biodegradable and negatively impacts plant growth by reducing photosynthetic pigments, leading to lower productivity [11]. The researchers found that higher levels of Cr accumulation in plants hinder germination and stunt the roots and shoots growth, which eventually affects total biomass and yield [12]. Gill et al. in (2016) noted a similar pattern, observing diminished seedling growth and biomass in *Brassica napus* cultivars subjected to Cr concentrations ranging from 0 to 400 µM [12]. Numerous studies have described the effects of Cr on plant growth, biomass, and chlorophyll (Chl) content (both Chl a and b, as well as total), in addition to its toxicity to enzyme function [13–17]. Furthermore, excessive Cr induces oxidative stress in different plant tissues by increasing the generation of reactive oxygen species (ROS), hydrogen peroxide (H2O2), and malondialdehyde (MDA), along with raising electrolyte leakage (EL) [18]. The oxidative stress caused by Cr leads to significant phytotoxic impacts in plants through ROS production at the cellular level which results in the oxidation of proteins and lipids, damage to nucleic acids, and inhibition of enzymes, causing harm to cellular components and eventually leading to cell death [19,20].

Chickpea (*Cicer arietinum* L.) also known as Channa (common name), is an important crop in Pakistan mostly cultivated in Punjab and Sindh with a cultivation area of 2.2 million ha and a significant role in the GDP of the agricultural sector of the Pakistan’s economy [21]. Despite its significance, chickpea cultivation experiences climatic and edaphic challenges particularly heavy metals (Cr) contamination in soil near industrial units. As chickpea is Pakistan’s most widely grown pulse crop, contributing significantly to national protein intake; thus, Cr-induced reductions in its productivity pose direct risks to food and nutritional security. Due to their well-adaptivity to local conditions, the selected varieties for this study (CM-98 and CM-72) are mostly cultivated in Pakistan [21]. However, no comprehensive study has been carried out to assess the impacts of Cr on chickpea in Pakistan. Previous studies have revealed the Cr tress impacts on plant growth, chlorophyll content, nutrient uptake, and yield in crops but this remains unexplored in Pakistani chickpea cultivars. Therefore, this is the first study to examine Cr(VI) effects on germination behavior and early seedling development of Pakistani chickpea varieties, providing foundational baseline data for future physiological, biochemical, and breeding studies.

## Materials and methods

### Plant materials

**Fig 1:**
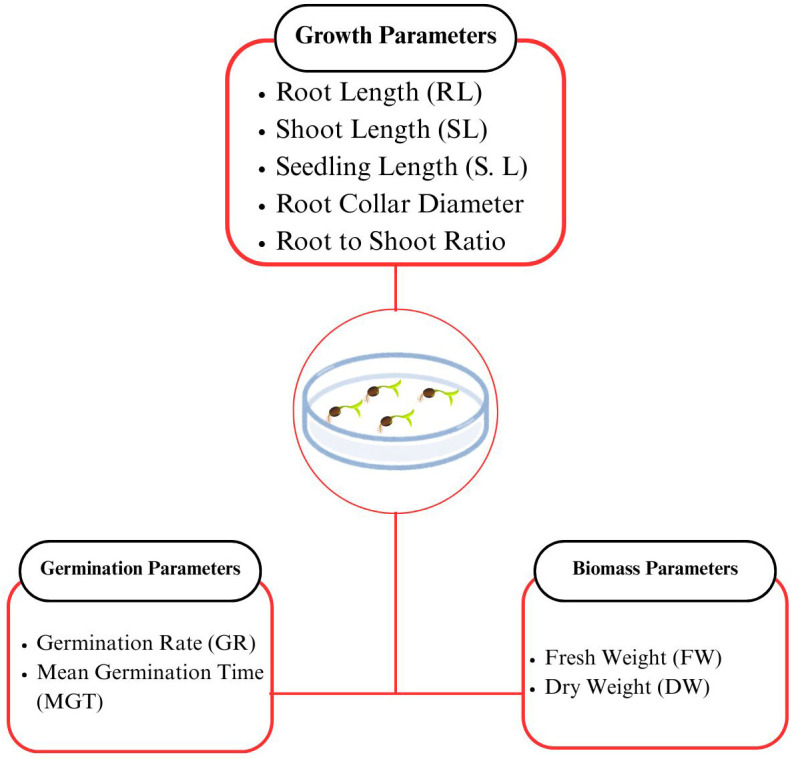
Experimental parameters measured during the assessment of chromium toxicity on chickpea (*C. arietinum* L.) germination and seedling establishment. Data collected included germination metrics, growth characteristics, and biomass accumulation.

Two chickpea Varieties, CM-72 and CM-98 were received from Arid Zone Research Institute (AZRI), Bhakkar, Punjab, Pakistan. The equivalent size healthy seeds were selected for the germination in present study. All the seedling growth parameters assessed under Cr stress in the study are illustrated in Fig 1.

### Experimental design

**Fig 2:**
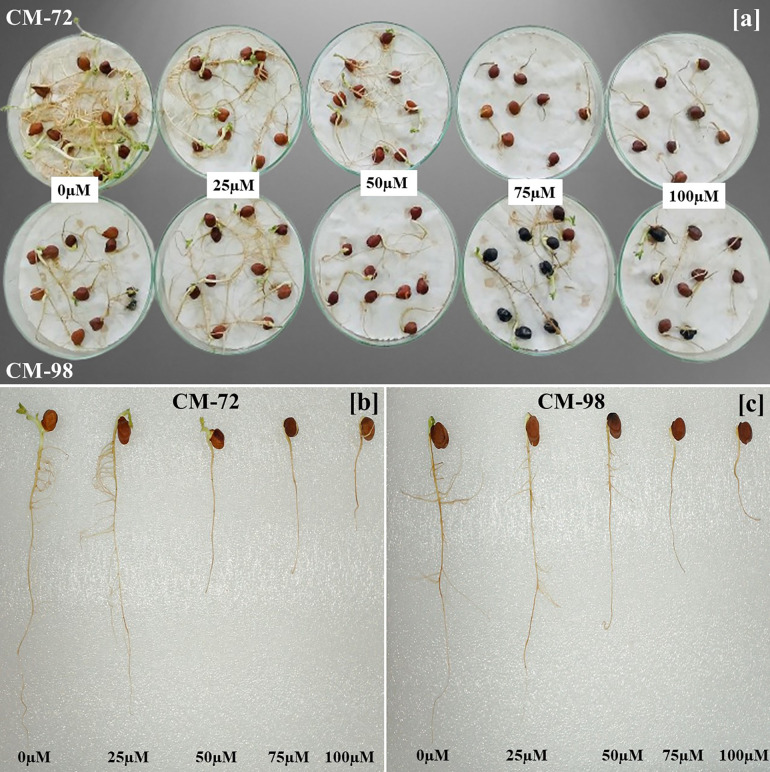
Photographic illustration of chromium toxicity effects on chickpea (*Cicer arietinum* L.). [a] Germination of varieties CM-72 (top) and CM-98 (bottom) at 0, 25, 50, 75, and 100µM Cr. [b, c] Corresponding representative seedling growth for CM-72 and CM-98, respectively.

A Completely Randomized Design (CRD) was employed for the investigation of this study. The experiment contained five chromium treatments, 0µM (control), 25µM, 50µM, 75µM, and 100µM by taking potassium dichromate (K_2_Cr_2_O_7_) as source. The same size healthy seeds were sterilized using a 0.2% solution of MgCl_2_ and then washed out with distilled water. MgCl₂ (0.2%) was selected because it is less phytotoxic than ethanol or sodium hypochlorite, minimizing potential pre-germination membrane injury and ensuring no confounding stress effects during chromium exposure. Prior to the germination, seeds were soaked in their respective chromium solutions, while a control was taken in distilled water for 24 hours. Subsequently, ten seeds were arranged in each petri dish lined with filter papers that were moistened with respective treatment solutions (Fig 2). Petri dishes were placed in a germination chamber and filter papers were not replaced to avoid seedling disturbance, but Cr (VI) solutions were reapplied as needed to sustain moisture and treatment integrity. No evaporation-induced concentration changes were observed. The germination chamber maintained 25°C, 10/14 h light/dark cycles, and 85–90% relative humidity throughout the trial to ensure that Cr(VI) was the sole stress factor. Germination parameters were assessed regularly every 24 hours for 14 days. Germinations were considered complete with the emergence of 2 mm of the radicle.

### Measurements

Seedling responses were analyzed based on final germination, growth and biomass parameters on the 14^th^ day by taking the average for each parameter from 5 randomly selected seeds per replica.

### Germination parameters

Germination rates (GR) were calculated following the method provided by the Association of Official Seed Analysts [22] using the formula:


$$GR=\frac{G}{S}\times \text{1}\text{0}\text{0}$$

Here, *G* is a number of seeds germinated while *S* is the total number of seeds sown for germination. Mean germination time was accessed using the calculation method employed by Ellis & Roberts in 1981 stated as follows [23]:


$$MGT=\frac{\Sigma (Ni\times Ti)}{\Sigma Ni}$$

Where *Ni* is a number of seeds germinated on day *Ti* which is the number of days from which the experiment started, *ΣNi* is the total number of seeds germinated and *i* indicates the day of scoring. Germination index was measured using the formula provided by the Association of Official Seed Analysts [24] stated as follows:


$$GI=\Sigma \frac{Gi}{Ti}$$

*Gi* indicates the number of seeds germinated on day *i* while *Ti* is the total number of days the seeds were sown.

### Growth and biomass parameters

Growth and biomass parameters like Root Length (RL), Shoot Length (SL), Seedling Length (S. L), Root collar diameter, root to shoot ratio and fresh weight, dry weight were measured by using a digital length measuring and weigh balance analyzer respectively.

### Statistical analysis

The results were analyzed by employing appropriate statistical methods to evaluate seedling responses of CM-72 and CM-98 to Cr stress. A two-way analysis of variance (ANOVA) was performed to interpret significant differences in Cr concentrations and two varieties for each parameter. To identify specific differences among treatment groups Post-hoc tests were employed. In addition to significance testing, effect sizes (η²) were also calculated for all ANOVA results to estimate the proportion of variance explained by each factor (S2 Table). Pearson’s correlation analysis was carried out to analyze the significant relationship among varieties for each parameter measured. Additionally, Principal Component Analysis was used to visualize multivariate analysis and specific responses of parameters. All the statistical and visualization methods were performed on R software, taking p value < 0.05, and results were presented as mean ± standard error (SE).

Before conducting the multivariate analysis, the assumptions for Principal Component Analysis (PCA) were also assessed. The Kaiser-Meyer-Olkin (KMO) statistic confirmed the adequacy of the sample size (KMO = 0.72), and Bartlett’s test of sphericity showed there was sufficient correlation for reducing dimensionality (χ² = 687.04, p < 0.001). The PCA identified two principal components (PC1 and PC2) that accounted for 61.5% and 22.9% of the total variance, respectively (S1 Table). A two-way ANOVA revealed significant impacts of Cr concentration and variety on all parameters (p < 0.001; S2 Table), with post-hoc tests validating dose-dependent differences (LSD, p < 0.05).

### Safety protocols

Cr (VI) waste was detoxified using sodium meta-bisulfite reduction, neutralized, and disposed via institutional hazardous waste protocols in compliance with local regulations.

## Results

### Germination parameters

**Fig 3:**
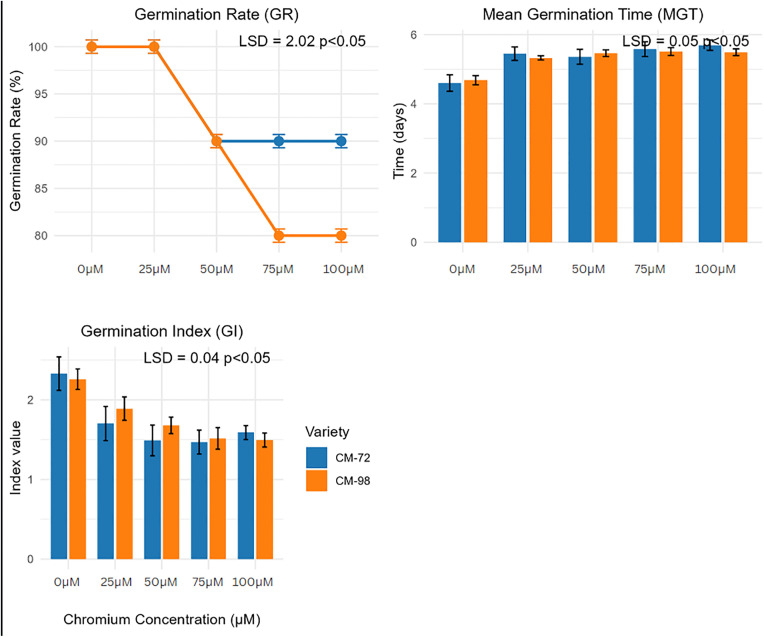
Effect of varying chromium concentrations (0, 25, 50, 75, 100 µM) on Germination Rate (GR, %), Mean Germination Time (MGT, days), and Germination Index (GI) of two chickpea (*Cicer arietinum* L.) varieties (CM-72 and CM-96). Values represent the mean ± standard error (SE). Least Significant Difference (LSD) values at p < 0.05 are indicated for comparing means within each parameter.

Significant variations were observed in seedling responses by both chickpea varieties under Cr stress for germination rate, mean germination time, and germination index. Both varieties showed a high germination rate (100%) at 0µM and 25µM. While at high concentration, CM-72 showed more tolerance by maintaining a 90% germination rate as compared to CM-98 which exhibited an 80% rate, indicating its greater sensitivity toward Cr stress. Similarly, a delayed mean germination time was examined as CM-72 showed an increase from 4.9 to days to 5.78 days, however, an increase of 4.8 to 5.5 days was observed for CM-98, which indicates metabolic inhibition induced by Cr stress. Additionally, germination index outcomes were also observed in a progressive decline manner as CM-72 showed a 2.13 index to 1.61, while a 2.16 index to 1.48 was observed for CM-98. A declined germination index suggests reduced germination efficiency of both Varieties against Cr stress. Fig 3 illustrates the germination responses by both Varieties to Cr stress.

### Growth parameters

**Fig 4:**
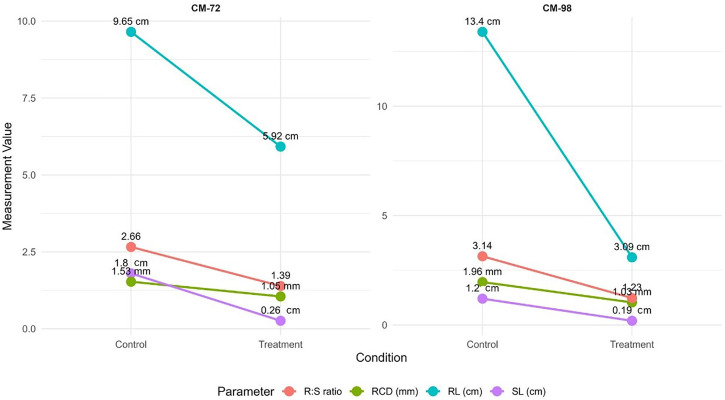
Influence of chromium treatment on key growth metrics (Root Length, Shoot Length, Root Collar Diameter, Root:Shoot ratio) in chickpea (*Cicer arietinum* L.). Left panel: Variety CM-72. Right panel: Variety CM-98. Mean values are shown for control (0µM) and treatment groups (100µM).

**Fig 5:**
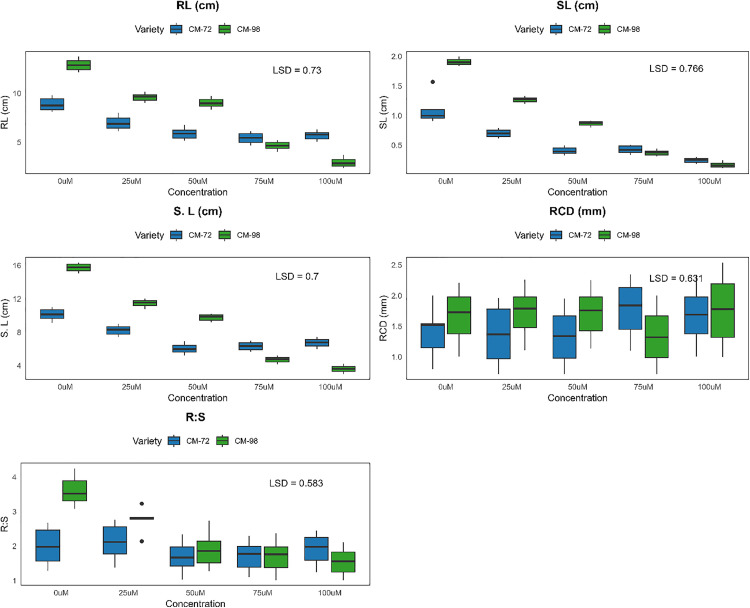
Variability and central tendency of key growth metrics (RL, SL, S.L, RCD, R:S) in chickpea (*Cicer arietinum* L.) varieties CM-72 and CM-98 under chromium stress (0-100 µM). Boxplots visualize the distribution for each parameter, variety, and concentration. LSD values (p < 0.05) facilitate comparison of means. (Units as indicated on axes).

Cr stress notably affected the root and shoot development resulting in impaired seedling growth in both chickpea Varieties (Fig 4). A dose-dependent decline was exhibited by both Varieties with the increasing concentration in root length as CM-98 showed a greater sensitivity with the 77% decline from 13.04 cm (0µM) to 3.09 cm (100µM) while a lower but consistent 39% decline from 9.65 cm (0µM) to 5.92 cm (100µM) was shown by CM-72. Similarly, 90% reduction was observed in shoot length by CM-98 as compared to CM-72 which exhibited 76% reduction from 1.8 cm to 0.26 cm (0µM −100µM). Additionally, a 79% reduction was experienced in CM-98 while CM-72 had with 42% reduction in seedling length (15.32 cm-3.28 cm) and (10.73 cm-6.18 cm) respectively. Higher Cr concentration significantly reduced the root collar diameter in both varieties as 47% and 31% reduction was exhibited by CM-98 and CM-72 respectively. The reduced impact of Cr(VI) on CM-72 root collar diameter may reflect structural reinforcement (lignification) or comparatively slower Cr translocation to basal stem tissues, which has been reported in tolerant chickpea and legume genotypes [14]. Subsequently, a reduction of 61% and 48% in both CM-98 and CM-72 respectively for root-to-shoot ratio indicated the greater root sensitivity of both Varieties to Cr stress. Variations in growth matrices under Cr stress are illustrated in Fig 5.

### Biomass parameters

**Fig 6:**
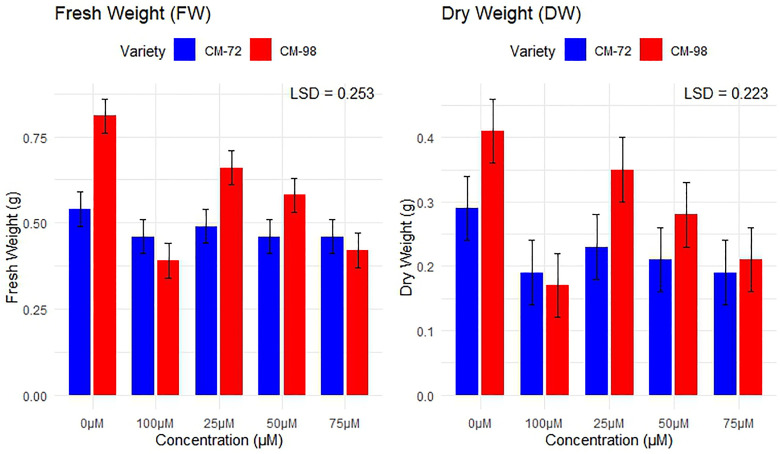
Seedling biomass accumulation, measured as Fresh Weight (FW, g) and Dry Weight (DW, g), in two chickpea (*Cicer arietinum* L.) varieties (CM-72 and CM-98) subjected to varying chromium concentrations (0-100 µM). Data represent mean ± SE, with LSD values (p < 0.05) indicated for statistical comparison of means.

Increasing Cr concentration negatively affected the biomass related parameters of both varieties with strong impacts on fresh and dry weight (Fig 6). At 100µM, fresh weights were reduced by 52% (0.81 g to 0.39 g) and 15% (0.54 g to 0.46 g) in CM-98 and CM-72 respectively, while comparing with results of control. The same pattern was followed by dry weight, a reduction of 59% (0.41 g to 0.17 g) and 34% (0.29 g to 0.19 g) by CM-98 and CM-72 respectively was experienced in comparison to the control. Both varieties exhibited a significant decrease in biomass production at higher Cr concentration.

### Multivariate correlation analysis

**Fig 7:**
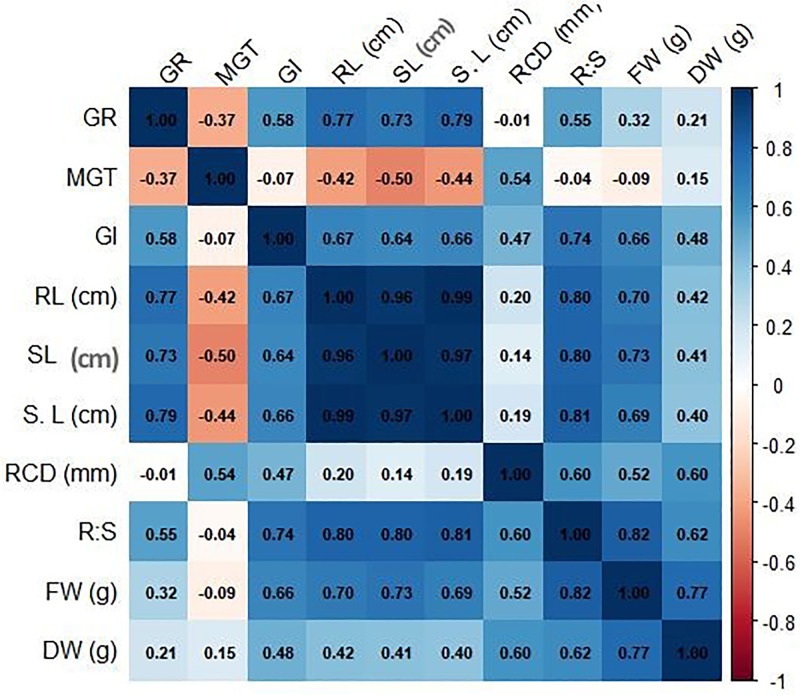
Correlation analysis of chickpea (*Cicer arietinum* L.) seedling traits. The heatmap shows Pearson correlation coefficients (r) between all pairs of measured parameters, including germination, growth, and biomass metrics (units as indicated). Color intensity reflects the strength of the correlation.

Pearson correlation significantly highlighted the germination, growth and biomass parameters under Cr stress (Fig 7). Root length (RL), shoot length (SL), and seedling length (S. L) showed a strong positive correlation (r > 0.90, p < 0.001) which indicates accompanying growth inhibition in these traits. Additionally, germination rate (GR) also shows positive correlation with RL (r = 0.77) and SL (r = 0.73), indicating better early seedling growth in seeds with higher germination index (GI) despite Cr stress. However, a negative correlation was observed in mean germination time (MGT) with GP% (r = −0.37) and growth traits (r = −0.42 to −0.50), suggesting that delayed MGT resulted from Cr-induced growth suppression. An inter-correlation was also observed within biomass parameters (r = 0.77), and between fresh weight (FW) and root to shoot ratio (R:S) (r = 0.82), which indicated combined structural and metabolic impacts of Cr stress.

**Fig 8:**
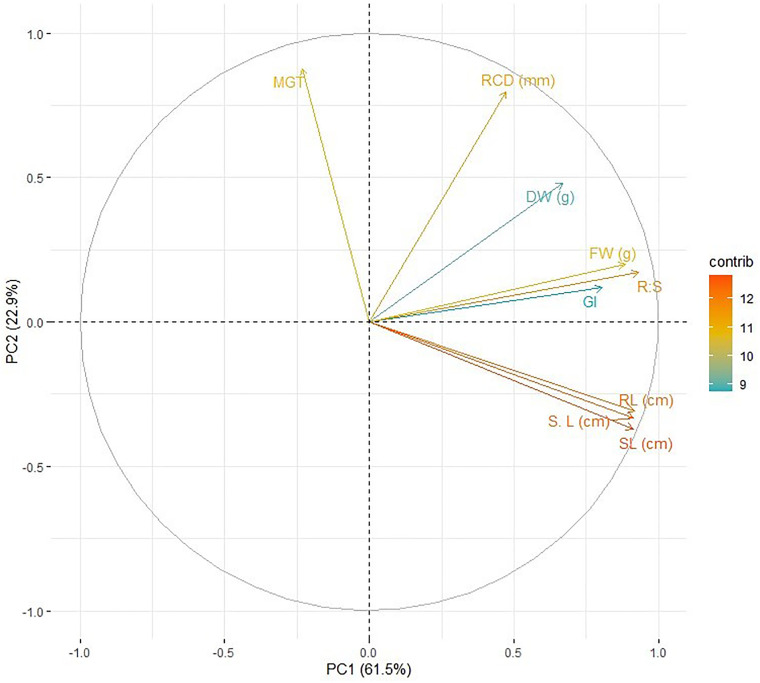
Principal Component Analysis (PCA) biplot illustrating the relationships between measured chickpea (*Cicer arietinum* L.) parameters and their contribution to the main axes of variation (PC1: 61.5%, PC2: 22.9%). Vector positions reveal correlations among variables and their association with the principal components. Color indicates contribution strength (‘contrib’).

Furthermore, principle component analysis (PCA) reveals relationship between growth (RL, SL, S. L) and biomass traits (FW, DW) with PC1 (61.5% variance), however, MGT and root collar diameter (RCD) are separated from other variables with 22.9% variance (PC2) (Fig 8). This separation indicates that Cr stress independently affected the growth (PC1), MGT, and root structural modifications (PC2). Additionally, GI clustering with PC1 indicates its role as a seedling performance predictor under stress. PCA differentiated germination-related traits (e.g., MGT, GP) from early structural growth traits (e.g., root length, shoot length), indicating that Cr stress affected these trait groups differently.

## Discussion

The present study investigates the responses of germination and seedling development by two chickpea varieties (*Cicer arietinum* L.), CM-98 and CM-72 to Cr stress. The variations observed in germination, growth and biomass indices under increasing Cr concentration indicates differential genotypic Cr tolerance. As both varieties shown reduced seedling responses to increasing Cr stress, although CM-72 demonstrate more tolerance as compared to CM-98, which aligns with the findings of Singh et al. (2020), who reported these results in chickpea varieties, Pusa 2085 which shown better growth and higher tolerance against Cr stress compared to Pusa Green 112 [15]. Cr-induced reduced germination is due to poor water uptake, enzymatic and energy metabolism disruption which are the main reasons for heavy metals-induced stress [12].

CM-98 showed higher sensitivity to Cr-stress as it exhibited 77% and 90% decline in root and shoot length, respectively, under 100µM. However, the reduction in CM-72 was quite moderate, which emphasized its greater tolerance to Cr stress. A similar finding identified seedling growth-limiting mechanisms such as cellular elongation disruption and mitotic activity inhibition [25]. Moreover, results of Singh et al. (2020) were similar to present study as Pusa Green 112 shown more sensitivity to >90µM which mirror to results of CM-98 of this study [15]. Findings on biomass parameters in the present study underscore differential tolerance as CM-98 exhibited a sharp decline in both fresh and dry weights, particularly at higher Cr concentration. These results emphasized the catastrophic impacts of Cr stress on metabolic ability and structural integrity which are similar to prior studies carried out on decreased chlorophyll content, membrane, and enzymatic disruption induced by Cr stress [26,27].

Furthermore, multivariate analysis elaborates stress-induced traits associations as Principal Component Analysis clearly emphasized and distinguished growth and biomass traits along PC1 and root collar diameter and mean germination time on PC2. This separation in the present study aligns with the findings of Singh et al. (2020), who reported it in enzymatic responses and proline accumulation in dose-dependent differentiation [15].

This study supports the proposed defense mechanism against Cr stress, such as enhanced proline accumulation, antioxidant activity, and reduced electrolyte leakage, which were observed in the more tolerant variety Pusa 2085 [15], which suggests specific tolerance mechanisms in CM-72 against Cr toxicity. Subsequently, dose-dependent effects observed across all parameters also support the results reported in legume studies, such as *Vigna unguiculata* and *Brassica napus,* which showed similar responses for germination, growth, and biomass parameters [28,29]. These consistent results against Cr stress emphasize the general account of Cr phytotoxic impacts and the more tolerant varieties for better crop production.

## Conclusion

Growing prevalence of chromium (Cr) in various industrialized sectors has led to significant environmental pollution over time which negatively impacts crops, including chickpeas. Cr exposure also detrimentally affects seed germination and the morpho-physiological and biochemical traits, such as plant growth, chlorophyll content, nitrogen content, and germination potential of chickpeas. The harmful effects stem from heightened bioaccumulation of Cr, resulting in heavy metal stress. Specifically, Cr stress increases the production of malondialdehyde (MDA) and hydrogen peroxide (H_2_O_2_), as well as electrolyte leakage, while decreasing total protein levels in chickpea seeds. In this research, the enhanced early-stage tolerance of CM-72 indicates its possible suitability for growth in chromium-affected areas; however, it is crucial to conduct field trials throughout various growth phases to verify its agricultural feasibility and yield consistency under prolonged stress. These baseline findings offer a starting point for developing Cr-resilient cultivars, supporting sustainable agriculture in metal-contaminated regions and informing future chickpea breeding programs. Future biochemical analyses should quantify antioxidants such as superoxide dismutase (SOD) and proline, which are commonly elevated in Cr-tolerant legumes and may underlie the observed tolerance of CM-72. Identifying molecular markers (e.g., SNPs linked to Cr-tolerance traits) in CM-72 would also facilitate marker-assisted selection for breeders working to develop Cr-resilient chickpea varieties. Nevertheless, to gain a thorough understanding of the tolerance mechanisms to Cr toxicity, it is essential to further investigate the interactions among these processes at the biochemical and genetic level across various crops. Such research could aid in managing stress induced by heavy metals in crops, which is becoming an increasingly pressing issue in agricultural systems.

## Supporting information

S1 Table PCA diagnostics and variance explained by principal components. (DOCX)

S2 Table Two-way ANOVA results for germination, growth, and biomass parameters with Effect size (η²). (DOCX)
