# Correction: Differential seedling responses of chickpea varieties to hexavalent chromium (VI) stress under controlled conditions

**DOI:** 10.1371/journal.pone.0348897

**Published:** 2026-05-05

**Authors:** Muhammad Hammad, Afifa Kainat Rani, Laraib Chouhdary, Muhammad Kabir, Patricio R. De los Rios-Escalante, Muhammad Aslam

There is an error in affiliation 4 for author Patricio R. De los Rios-Escalante. The correct affiliation 4 is: Departamento de Ciencias Biológicas y Químicas, Facultad de Recursos Naturales, Universidad Católica de Temuco, Chile.
