# Supplementary material for: Differential seedling responses of chickpea varieties to hexavalent chromium (VI) stress under controlled conditions
Source: PLoS One. 2026 Jan 29;21(1):e0341546. doi: 10.1371/journal.pone.0341546 (PMC12854415; doi:10.1371/journal.pone.0341546)
Supplement: S1 Table — (DOCX) [file pone.0341546.s001.docx]

**Table S1.** PCA diagnostics and variance explained by principal components.

| **Component** | **Standard Deviation** | **Proportion of Variance** | **Cumulative Proportion** |
| --- | --- | --- | --- |
| PC1 | 2.45 | 61.5% | 61.5% |
| PC2 | 1.49 | 22.9% | 82.3% |
