# Supplementary material for: Differential seedling responses of chickpea varieties to hexavalent chromium (VI) stress under controlled conditions
Source: PLoS One. 2026 Jan 29;21(1):e0341546. doi: 10.1371/journal.pone.0341546 (PMC12854415; doi:10.1371/journal.pone.0341546)
Supplement: S2 Table — (DOCX) [file pone.0341546.s002.docx]

**Table S2.** Two-way ANOVA results for germination, growth, and biomass parameters with Effect size (η²).

| **Parameter** | **DF** | **F_value** | **p_value** | **η² (Variety)** | **η² (Concentration)** |
| --- | --- | --- | --- | --- | --- |
| GR | 9 | 124.4444444 | p <0.001 | 0.0690 | 0.6983 |
| MGT | 9 | 5.2581890 | p <0.001 | 0.0020 | 0.3718 |
| GI | 9 | 4.3647809 | p <0.001 | 0.0036 | 0.3546 |
| RL | 9 | 119.7599052 | p <0.001 | 0.0491 | 0.6811 |
| SL | 9 | 145.5045381 | p <0.001 | 0.1205 | 0.7111 |
| SL | 9 | 145.5045381 | p <0.001 | 0.0511 | 0.6581 |
| RCD | 9 | 7.183383 | p <0.001 | 0.0184 | 0.0135 |
| RS | 9 | 7.8505803 | p <0.001 | 0.0796 | 0.2964 |
| FW | 9 | 6.4586415 | p <0.001 | 0.2975 | 0.2443 |
| DW | 9 | 2.1888680 | p <0.001 | 0.1130 | 0.0665 |
